# Electrografting of 4-Nitrobenzenediazonium Salts on Al-7075 Alloy Surfaces—The Role of Intermetallic Particles

**DOI:** 10.3390/nano11040894

**Published:** 2021-03-31

**Authors:** Jiangling Su, Juan Carlos Calderón Gómez, Guido Grundmeier, Alejandro González Orive

**Affiliations:** 1 Department of Technical and Macromolecular Chemistry, Paderborn University, Warburger Str. 100, 33098 Paderborn, Germany; sujl@mail.uni-paderborn.de (J.S.); guido.grundmeier@uni-paderborn.de (G.G.); 2 Department of Chemistry, Materials and Nanotechnology Institute, University of La Laguna, 38200 San Cristóbal de La Laguna, Spain

**Keywords:** aluminium alloy, aryldiazonium salts, electrografting, surface, intermetallic particles

## Abstract

In this work, the electrografting of Al-7075 aluminium alloy substrates with 4-nitrobenzenediazonium salt (4-NBD) films was studied on a complex aluminium alloy surface. Prior to the electrografting reaction, the substrates were submitted to different surface treatments to modify the native aluminium oxide layer and unveil intermetallic particles (IMPs). The formation of the 4-NBD films could be correlated with the passive film state and the distribution of IMPs. The corresponding electrografting reaction was performed by cyclic voltammetry which allowed the simultaneous analysis of the redox reaction by a number of complementary surface-analytical techniques. Spatially resolved thin film analysis was performed by means of SEM-EDX, AFM, PM-IRRAS, Raman spectroscopy, XPS, and SKPFM. The collected data show that the 4-NBD film is preferentially formed either on the Al oxide layer or the IMP surface depending on the applied potential range. Potentials between −0.1 and −1.0 V_Ag/AgCl_ mostly generated nitrophenylene films on the oxide covered aluminium, while grafting between −0.1 and −0.4 V_Ag/AgCl_ favours the growth of these films on IMPs.

## 1. Introduction

Aluminium alloys of the 7000 series are widely recognised due to their high strength, promoting their favourable application in automotive lightweight constructions [1]. This material is constituted by an aluminium matrix embedding intermetallic particles (IMPs) with sizes between 5 and 10 µm, which are composed of metals such as iron, copper, zinc, and magnesium [2,3]. It is well known that these IMPs have variable composition, a fact that promotes a differential activity on the surface of these alloys. For instance, in the case of the alloy Al-7075-T651, the compositions Al_2_CuMg, Mg_2_Si, Al_2_Cu, Al_7_Cu_2_Fe, (Al,Cu)_6_(Fe,Cu), and Al_3_Fe have been reported as the most common within the intermetallic particles [4,5]. These IMPs can be exposed on the surface of the alloy after applying chemical etching procedures, due to the dissolution of the native aluminium oxide layer, a process that is favoured at the borders of the IMPs [6]. The etching procedures also alter the roughness of the surfaces, changing its surface energy depending on the applied treatment. For instance, Guo et al. found an increase in the roughness of Al alloy 2024 after applying chemical etching involving NaOH solutions with concentrations higher than 4%, whereas negligible surface changes were observed at NaOH concentrations lower than this value [7].

The aluminium matrix spontaneously forms a protective insulating ultra-thin oxide film with terminating surface hydroxyl groups under atmospheric conditions and near neutral pH-values [8,9]. Such hydroxylated aluminium oxide surfaces enable the stable binding of molecules with corrosion protecting and/or adhesion promoting properties such as organophosphonic acids [10,11,12,13,14], fatty acids [15,16,17], and organosilanes [18,19]. Aryl diazonium salts have been also reported as precursors of thin organic films adsorbed on aluminium alloys, with potential applications for the corrosion protection of these materials [20,21]. These organic salts can be electrografted on the surface of different metals to obtain films at the level of monolayers or multilayers, depending on the experimental conditions employed during the preparation. In fact, once a monolayer is electrografted on the surface, successive layers can be subsequently attached by means of the attack of freshly formed aryl radicals, increasing thus the thickness of the film [22]. The electrografting via electroreduction of diazonium salts is often performed on metal/metal oxide surfaces by dissolving this compound in acetonitrile or from acid/neutral aqueous solutions. Cathodic potentials are applied by cyclic voltammetry to determine the reduction potential for the diazonium salt on the studied surface. This reduction potential is consequently used for the aryl-based film growth by means of potentiostatic deposition or by selecting a suitable potential range for cycling [22].

To date, many reports have dealt with the electrografting of aryl-based moieties on different conductive materials: carbon substrates such as glassy carbon [23,24,25], highly-oriented pyrolytic graphite [26,27,28], pyrolysed photoresist film (PPF) [29] and noble metals such as Au [30,31] and Pt [32]. However, much less effort has been made regarding easily oxidisable metals like copper [20], iron [33,34,35], zinc [36], nickel [37,38], ITO [39,40,41], chromium [42] and Al nanoparticles [43]. More recently, Berisha et al. have carried out the electrografting of alkyl iodides in the presence of a diazonium salt on pure aluminium, obtaining mixed alkyl/aryl films [44]. Some other reports have made use of this electrochemically induced deposition of organic layers to modify the surface properties of metallic substrates with industrial interest such as TiO_2_ [45], SiO_2_ [22], ZnO [36], NiTi alloys [46] or steel [47,48]. Nevertheless, to date no abundant investigations have addressed the electrografting of organic films via reduction of diazonium salts onto technically relevant aluminium alloys. Therefore, this fact would imply that the nature of the electrografting reaction is still unknown in terms of the active sites present in the surface of these alloys, which could play a key role in the formation of a stable film. In this regard, Hurley et al. deposited phenylene layers on the surfaces of Cu and the 2024 T3 aluminium alloy in order to suggest a novel alternative to reduce the corrosion processes on these metal surfaces, replacing in this way the chromium frequently used as cathodic inhibitor [20]. The authors found that a stable film can be built by means of the formation of both Cu–O–C and Cu–C bonds after the electrografting procedure. In the case of the aluminium alloy surface, a high concentration of the electrografted molecule was detected by Raman spectroscopy, suggesting that the molecule is deposited on both the aluminium oxide layer and the other low concentration elements. From a first point of view, it could be inferred that the minor components in the alloy did not exhibit a differential behaviour in terms of activity toward the deposition of the phenylene layer. However, it is noteworthy to mention that the presence of second phase intermetallic particles has a significant impact on the properties of these Al alloys, since its arising may cause a detriment in the mechanical properties of the Al alloy such as fracture hardness and fatigue resistance [49]. On the other hand, IMPs can be cathodic or anodic with regard to the aluminium matrix: a higher content in more noble elements like iron and copper or more active such as zinc or magnesium would result in cathodic or anodic particles, respectively. Consequently, the occurrence of these localised spots with either microanodic or microcathodic behaviour would result in galvanic reactions promoting pitting and crevice corrosion [5]. For these reasons, the fundamental understanding of the surface reactivity and interfacial properties of such intermetallic constituents is crucial for the preparation of lightweight material significantly free of defects and failures.

Considering the facts mentioned above, in the present work the electrografting of the aluminium alloy 7075 (Al-7075) with a 4-nitrobenzenediazonium salt (4-NBD) is studied, in order to find the active sites for the deposition of this molecule and the factors playing a key role in this process. 4-NBD was selected as a model grafting molecule widely reported in the literature and the possibilities it offers to be detected by means of spectroscopic and electrochemical techniques. The aluminium alloy surfaces were modified to unveil the intermetallic particles and to decrease the native passive film thickness. The electrografting of the diazonium salt was performed on selected surface states employing different potential ranges. To the best of our knowledge, the differential behaviour regarding the grafting process of 4-NBD onto the aluminium/aluminium oxide matrix and the embedded intermetallic particles is thoroughly discussed herein for the first time. The as-prepared films were characterised by different physical and electrochemical techniques in order to verify its successful deposition and the active sites where they are electrografted.

## 2. Materials and Methods

### 2.1. Surface Preparation of the Al-7075 Surfaces

The 10 mm × 10 mm × 3 mm-Al-7075 T6 (5.6 wt % Zn, 1.6 wt % Cu, 2.5 wt % Mg, 0.5 wt % Fe, Rasch Metalle GmbH&Co, Bielefeld, Germany) specimens were submitted to three different surface treatments as follows: (1) for the solvent cleaning treatment (SC), the Al-7075 samples were rinsed with three different solvents during 15 min under ultrasound radiation, in order to remove oil and grease residues from the surface. These solvents were tetrahydrofuran (THF, anhydrous, ≥99.9%, Sigma-Aldrich, St. Louis, MO, USA), isopropyl alcohol (≥99.7%, Sigma-Aldrich) and ethanol (≥99.8%, Sigma-Aldrich). Subsequently, the substrates were dried with nitrogen. (2) For the etched solvent cleaned substrates (ESC) the SC substrates were chemically etched with a 2 wt. % NaOH (≥97%, Sigma-Aldrich) solution during 30 s and then with a 10 wt. % (NH_4_)_2_S_2_O_8_ (≥ 98%, Sigma-Aldrich) solution for 10 s. (3) For the etching ion polish treatment (EIP), the solvent cleaned substrates were polished until achieve a mirror quality surface with SiC abrasive paper (grit 999, Klingspor GmbH&Co, Haiger, Germany), followed by polishing with 0.3-µm alumina powder in aqueous suspension (200 g·L^−1^, Schmitz GmbH&Co, Bünde, Germany). Then, the mirror-polished substrates were chemically etched as described for the ESC samples, rinsed, and dried. To obtain ion-polished surfaces, the substrates were submitted to a treatment with an Ar^+^ ions beam by means of an EM TIC 3X ion polisher (Leica Mycrosystems, Wetzlar, Germany). The substrates were put into a rotary stage under a maximum pressure of 10^−3^ Pa. Afterwards, the chamber containing the rotary stage was purged with Ar until achieve a pressure close to 5.0 × 10^4^ Pa. The Ar^+^ ions beam was generated from three guns operated with an acceleration voltage of 6 kV and a gun current of 2.2 mA during 3 h.

### 2.2. Electrografting of 4-NBD on the Al-7075 Substrates

The 4-nitrobenzene diazonium salt (4-nitrobenzenediazonium tetrafluoroborate used as received, 97%, Sigma-Aldrich) was electrografted on the treated Al-7075 substrates by means of a three electrode-cell, with an Ag/AgCl reference electrode (Radiometer Analytical, Hach Co. Loveland, CO, USA) and a gold wire-counter electrode. The freshly prepared Al-7075 substrates were employed as working electrodes displaying a geometrical area of 0.1964 cm^2^. The medium to perform the electrografting was a 2 mM 4-NBD solution in acetonitrile (ACN), containing 0.1 M tetrabutylammonium tetrafluoroborate as supporting electrolyte (TTBATFB_4_, >99%, Sigma-Aldrich). This solution was bubbled with nitrogen to remove the dissolved oxygen. The electrografting of the substrates was performed by cyclic voltammetry, applying a potential scan between −0.1 and −1.0 V vs. Ag/AgCl at 50 mV·s^−1^. Another grafting was done applying a shorter potential range, which considered a maximum negative vertex potential of −0.4 V. After the grafting processes, the 4-NBD modified substrates were thoroughly rinsed with ACN and subsequently ultrasonicated in ACN for 5 min and dried under N_2_ stream. The as-prepared films were employed for the characterisations described in further sections.

### 2.3. Characterisation of the Al-7075 Surfaces and 4-NBD Electrografted Films

#### 2.3.1. Scanning Electron Microscopy and Energy Dispersive X-ray Mapping (SEM-EDX)

SEM images of the bare substrates and the electrografted films were obtained by means of a NEON 40 FE-SEM microscope (Carl Zeiss SMT AG, Oberkochen, Germany), which was equipped with an InLens detector and a SE2. The EDX mapping was performed using different probe forming convergence semi-angles, which were taken each 35°, with the probe voltage set to 4 kV. The aperture was fixed to 120 µm, whereas the size of the frame was 256 × 192 pixels. In total, 40 ms were necessary to record each pixel.

#### 2.3.2. PM-IRRAS Analysis

The IR spectra of the different samples were obtained by means of a Vertex V70 PM-IRRAS (Bruker Optics GmbH, Leipzig, Germany) with a ZnSe photoelastic modulator (PMA50, Bruker Optic GmbH, Germany). The spectra were recorded and corrected by means of the software OPUS^®^ (version 7.8, Bruker Optic GmbH, Ettlingen, Germany). In each measurement, 512 scans were performed to record the spectra in air, with a resolution of 4 cm^−1^ in the wavenumber range of 800–4000 cm^−1^.

#### 2.3.3. Raman Spectroscopy

The Raman spectra of the samples were obtained by an InVia Renishaw confocal Raman microprobe system (Renishaw RE 04plc, Gloucestershire, UK) equipped with a Leica DM 2500 M confocal microscope and a *λ* = 532 nm laser.

#### 2.3.4. X-ray Photoelectron Spectroscopy

The X-ray photoelectron spectroscopy (XPS) analysis was performed with an Omicron ESCA+ system (Omicron NanoTechnology, Taunusstein, Germany), coupled to a hemisphere analyser, at a chamber pressure of <5 × 10^−10^ mbar. A monochromatic Al-K_α_ X-ray (1486.7 eV) was employed with a spot diameter of 600 µm and a take-off angle of 30°. The calibration of the spectra was performed at the C 1s peak (285 eV) as the internal reference. For peak fitting, a Shirley background was chosen, and the peak fitting was performed using a peak shape consisting of a convolution of a Gauss (30%) and Lorentzian (70%) shape. For the quantification, relative sensitivity factors supplied from Omicron GmbH were implemented in the CASA XPS database (version 2.3.18, Casa Software Ltd., Wilmslow, UK).

#### 2.3.5. SKPFM Analysis

The Scanning Kelvin probe analysis was performed to determine the contact potential differences on the surface of the bare and modified substrates, according with the presence/absence of 4-NBD films and the intermetallic particles. An MFP-3D-SA atomic force microscope from Asylum Research was used to perform the measurements in the amplitude modulation (AM-KPFM) mode (two-pass technique). The microscope was operated inside an anti-noise box equipped with an anti-vibration table. The measurements were carried out using Pt-coated cantilevers (HQ:DPE-XSC11, 80 kHz, 2.7 N m^−1^, purchased from Mikromasch, Wetzlar, Germany), in areas of 50 × 50 µm^2^, and setting the lift scan height to 50 nm. The images were obtained with a resolution of 512 × 512 lines, an amplitude of 1 V and a scan rate of 0.1 Hz (5 µm s^−1^). No data flattening, smoothing, or inversion of the surface maps was carried out. Thickness values of the nitrophenylene layers included in the text are the consequence of the statistic assessment of cross section profiles carried out at different regions of three different but equivalently modified Al electrodes. The Volta potential images of Al-7075 alloy show only positive values since the Pt tip is biased, which is indicative of a less noble Al alloy matrix, i.e., it exhibits a more anodic behaviour than the Pt reference tip. Consequently, lower potential figures recorded in certain regions would mean cathodic character. Conversely, higher potentials would be indicative of enhanced anodic behaviour. A patterned reference sample containing regular Al, Si, and Au domains (as purchased from Bruker) was measured with analogue Pt tips under the same experimental conditions used herein for the characterisation of the Al alloys. The as-obtained SKPFM measurements are displayed in Figure S1. As expected, positive contact potential values, similar to those measured in the Al matrix of Al-7075, were recorded at the Al regions of the reference sample. Only slightly positive (or even negative) values were registered in the case of Au regions. Intermediate positive potential figures were recorded for the Si domains. A much more detailed explanation on the nobility, polarity, referencing, and other precautions when working with SKPFM are provided in the seminal contributions reported by Örnek et al. [50,51].

## 3. Results

### 3.1. Physical Characterisation of the Bare Substrates

Before the electrografting of the 4-NBD on the substrates, a SEM-EDX analysis of the Al-7075 surfaces after different surface treatments, namely solvent cleaning (SC), etched solvent cleaned (ESC) and etched ion polished (EIP), was performed. These results are presented in Figure S2 of the Supplementary Information. In general, similar intensities were found in all maps for each of the studied elements and the presence of intermetallic particles could be verified from the iron EDX maps. In the areas where these particles were observed, a decrease in the surface content of aluminium was also detected. Particularly, a slight increase in the Fe signal intensities could be appreciated in the treated substrates as a consequence of the intermetallic particles unveiling.

The bare substrates were also analysed by X-ray photoelectron spectroscopy in order to determine their surface composition according to the applied surface treatment. This composition was calculated from the area of the corresponding resolved spectra for each element. Table 1 presents these results, which displayed higher values for the atomic percentage of copper and magnesium in the case of the substrates submitted to the surface treatments, meaning that these procedures unveil the intermetallic particles. The unveiling of the intermetallic particles was also observed from the increase in the intensities observed for Fe in the EDX maps (see Figure S2 in the Supplementary Information) [2]. In general, the FE-SEM-EDX and XPS measurements unveiled the presence of intermetallic particles potentially able to form micro-galvanic cells with the aluminium alloy matrix [52]. Considering the XPS results displayed in the Table 1, a differential behaviour concerning the composition of the arising surface defects (barely present on the solvent-cleaned substrates) is observed: while the chemical etching favours the unveiling of both anodic and cathodic IMPs, most likely MgZn_2_ and Al_7_Cu_2_Fe, respectively [4,49], the ion polishing seems to mostly promote the development of cathodic IMPs.

**Figure 1:**
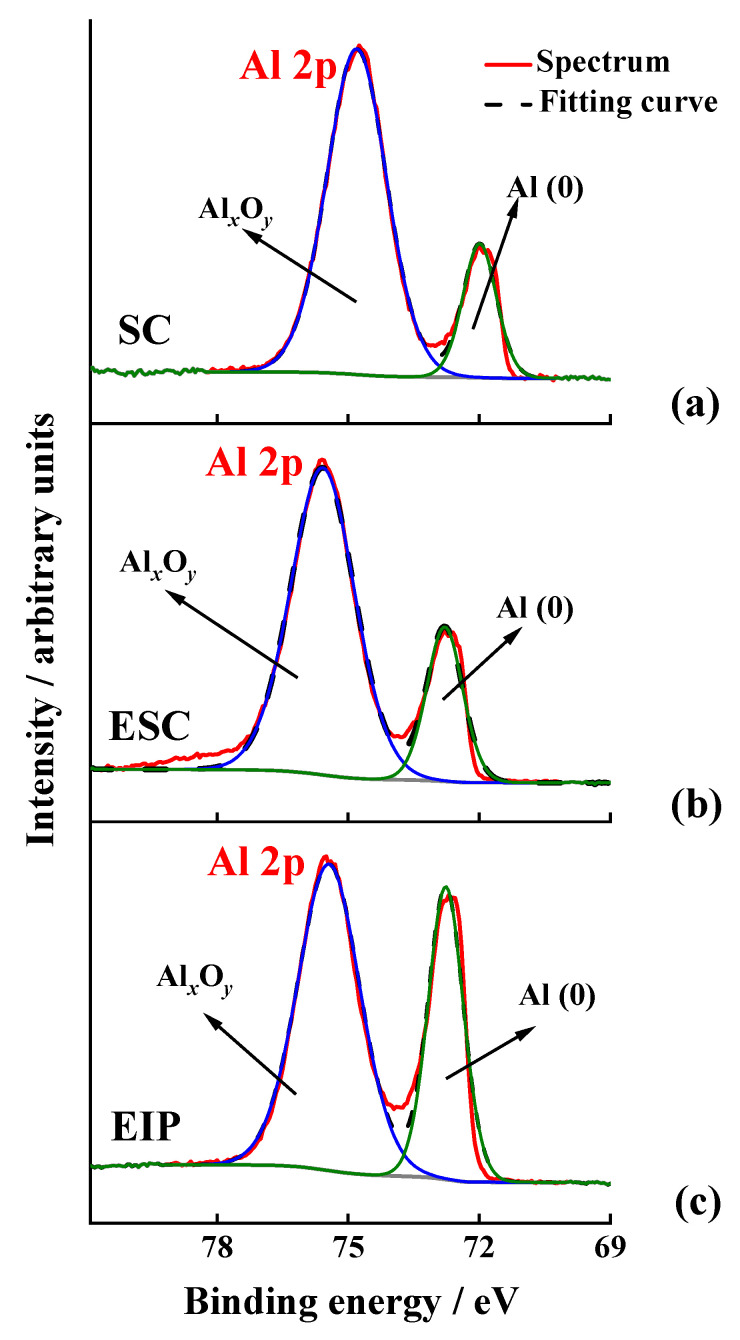
High resolution XPS spectra in the Al 2p region registered for the bare substrates after the (**a**) SC, (**b**) ESC and (**c**) EIP treatments.

The XPS spectra registered in the Al 2p region and collected for the different surface treatments of Al-7075 substrates are shown in Figure 1. The Al 2p peak shows two clearly differentiated components around 72 and 75 eV, attributed to metallic aluminium and aluminium oxide, respectively [53]. The position of the peaks for the treated ESC and EIP substrates (Figure 1b,c) is shifted toward higher binding energy values, a result that can be attributed to differences in the oxide growth process, possibly leading to different oxide thicknesses and structures [54]. In fact, an estimation of the aluminium oxide thickness *d* can be calculated from the relative intensities of the photoelectron peaks displayed in Figure 1, by means of the equation developed by Strohmeier (Equation (1)) [55]:

(1) $$d=\;\lambda_{Ox}\cdot sin\theta \cdot ln\left[\frac{N_{M}\cdot \lambda_{M}\cdot I_{Ox}}{N_{Ox}\cdot \lambda_{Ox}\cdot I_{M}}+1\right]$$

where *N_M_* and *N_OX_* are the volume densities of metal atoms in the metal (1.5) and the oxide (1.0), respectively. *I_M_* and *I_OX_* are the peak areas of the metal and the oxide, respectively, after removing the background. *λ_M_* and *λ_OX_* are the inelastic mean free paths of the photoelectrons (IMFP) from the metal (2.6 nm) and the oxide (2.8 nm), respectively [56]. On the other hand, θ is the photoelectron take-off angle (30°). The corresponding calculated layer thicknesses indicated that the oxide layer is decreased, especially in the case of the etched ion polish-treatment (see Table 2), matching with the trend reported by Hoppe et al. for surface treatments applied on aluminium surfaces such as pickling and mechanical brushing [57]. In summary, these results demonstrate that the chemical etching and the ion polishing treatments are useful to remove the Al oxide native layer. Furthermore, these results explain the EDX mapping intensities found for Fe in the treated substrates, which were higher than that observed for the SC substrate, as a consequence of an enhanced removal of the native Al oxide layer.

### 3.2. 4-NBD Electrografting of the Treated Al-7075 Substrates

**Figure 2:**
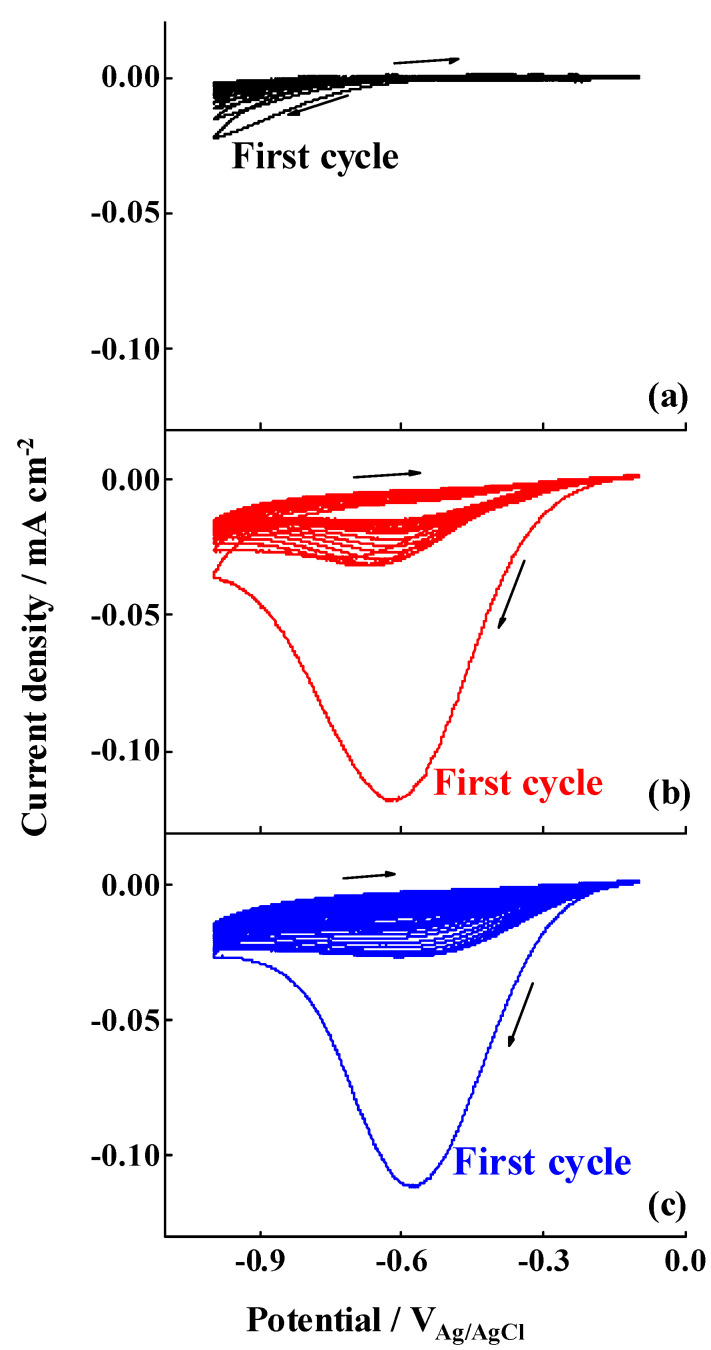
Cyclic voltammograms for the electrografting of 4-nitrobenzenediazonium salt (4-NBD) multilayers on (**a**) SC, (**b**) ESC and (**c**) EIP Al-7075 substrates employing a large potential range (−0.1 → −1.0 V_Ag/AgCl_). Supporting electrolyte: 0.1 M TBATTFB_4_ in acetonitrile. Scan rate: 50 mV·s^−1^.

After determining the morphological and chemical properties of the studied surfaces, the electrografting of the 4-nitrobenzenediazonium salt was performed by cyclic voltammetry, according to the procedure described in Section 2.2. The cyclic voltammograms registered when a large potential range is applied (−0.1 → −1.0 V) are shown in Figure 2. An irreversible diffusion-controlled voltammetric wave around −0.6 V (vs. Ag/AgCl) was observed in the first cycle for the case of the films electrografted on ESC and EIP-treated Al-7075 substrates. This peak is attributed to the electrochemical reduction of the diazonium salt, generating thus aryl radical species in the vicinity of the electrode that bind to the metal or oxide surface, upon release of N_2_, with the subsequent covalent attachment of nitrophenylene layers on the surface of the substrates. Similar results have been obtained for other metal oxide surfaces. For instance, those reported by Hinge et al. for the electroreduction of different diazonium salts electrografted on stainless steel [47] and by Adenier et al. for the electrografting of 4-NBD on iron electrodes [33]. Although, in these cases, the electrochemical reduction of the diazonium group occurs at slightly more positive values, the presence of a low conductive aluminium oxide layer would be responsible for the observed overpotential herein. Indeed, this conclusion is supported by the fact that the potential reduction is slightly less negative when the aluminium oxide layer is thinner, i.e., 1.8 nm, as occurred after the EIP procedure. In this regard, Hinge et al. carried out the electrografting of 4-NBD on a chromium electrode, covered with a thin film of native chromium oxide [42], which showed more negative values for the reduction of the diazo group as well. Nevertheless, the faradaic current densities are slightly higher in the case of ESC in comparison to those registered for EIP treated Al-7075 substrates. This can be attributed to a significant decrease in the roughness (electrochemical surface area) after ion polishing, as can be seen in Figure S3, since the data here presented are normalised by the geometrical area. Figure 2 also shows that, during successive cycling, the currents associated to this peak decreased as a consequence of the passivation of the surface with further nitrophenylene layers forming a compact film that inhibits the electronic transfer, thus avoiding the formation of more nitrophenyl radicals. In the case of the SC substrate (Figure 2a), featureless voltammograms were obtained and no peak was developed, a fact attributed to the presence of a thicker low-conductive aluminium oxide layer, i.e., 2.7 nm, as can be deduced from the XPS results shown in Table 2 that would inhibit, within the potential range selected, the formation of nitrophenyl radicals at the solid–liquid interface.

**Figure 3:**
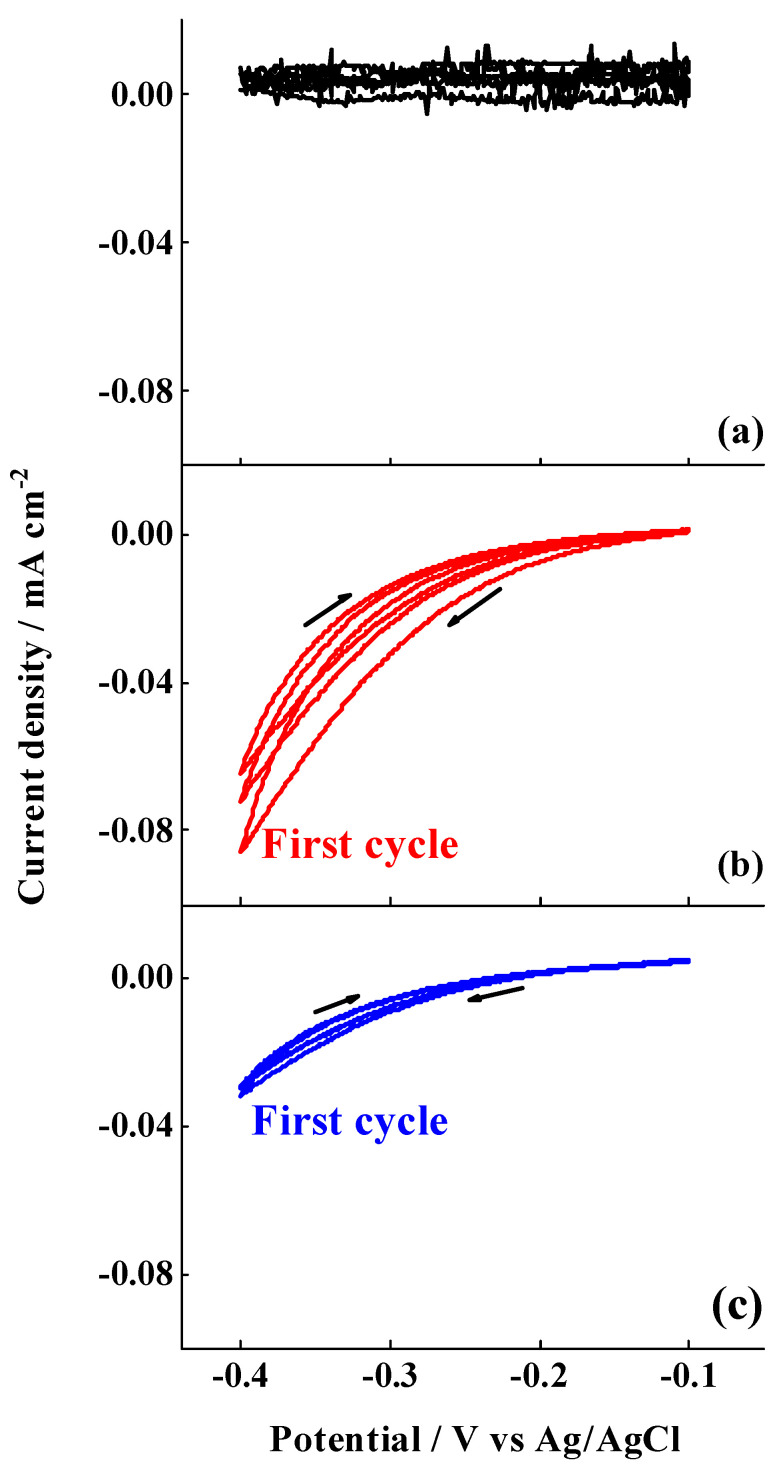
Cyclic voltammograms for the electrografting of 4-NBD multilayers on (**a**) SC, (**b**) ESC and (**c**) EIP Al-7075 substrates employing a short potential range (−0.1 → −0.4 V_Ag/AgCl_). Supporting electrolyte: 0.1 M TBATTFB_4_ in acetonitrile. Scan rate: 50 mV·s^−1^.

When the electrografting was performed applying a short potential range (−0.1 → −0.4 V_Ag/AgCl_), no diffusion-controlled reduction peaks were detected, as can be seen in Figure 3, although an increase in the current was observed in the case of the ESC and EIP-treated substrates when compared to SC Al-7075 substrates, suggesting the occurrence of a limited electrografting process despite the low negative potential values achieved in these experiments. Similar to the electrografting reaction performed by using the large potential range, there is a decrease in the current densities after successive cycling (the first three cycles are displayed in Figure 3), as a consequence of the progressive formation of a passivating film. In the case of the SC substrate, negligible current densities were registered, suggesting that no film was formed on this substrate.

### 3.3. SEM-EDX Analysis of the 4-NBD-Electrografted Al-7075 Substrates

**Figure 4:**
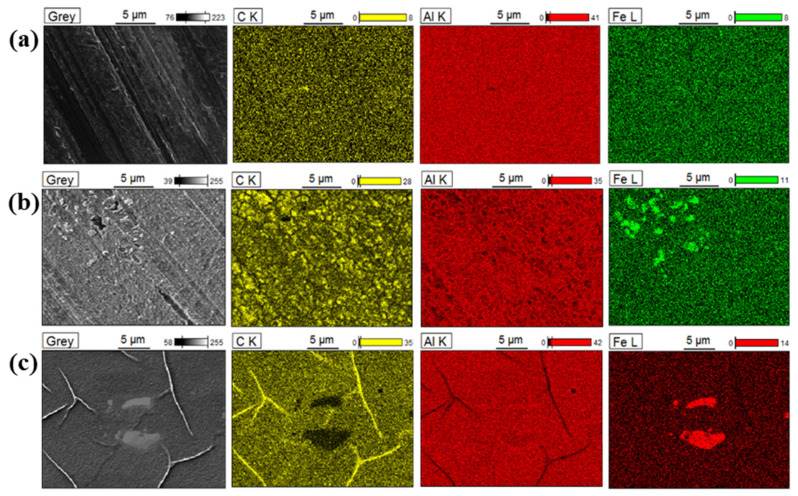
FE-SEM images and EDX maps of the 4-NBD electrografted substrates in the large potential range: (**a**) SC, (**b**) ESC and (**c**) EIP.

In order to characterise the morphology of the electrografted films obtained after cycling upon applying a large potential range, a FE-SEM-EDX analysis was carried out and these results are depicted in Figure 4. High intensity carbon EDX signals were observed after the 4-NBD electrografting process, particularly for the cases of the ESC and EIP-treated substrates, Figure 4b,c, whereas on the SC substrate a low intensity carbon signal was detected (see Figure 4a). This result indicates that the applied chemical etching and ion polishing treatments promote the formation of the nitrophenylene films, a fact that can be also verified from the values of the carbon and nitrogen percentage determined by EDX analysis after the electrografting by means of both, the large and the short potential range treatment, which are reported in the Table S1 of the Supplementary Information. The surface ratios of carbon and nitrogen varied for the electrografted substrates; a fact that could be explained by the formation of multilayers at different levels, as a consequence of the different characteristics of the substrates and the differences in the applied potentials. Furthermore, it seems that the electrografting was not particularly favoured on the surface of the intermetallic particles for all the studied substrates, considering that the low carbon EDX signal values match with the highest iron EDX signal values, an element that is abundantly present in the intermetallic particles. This effect is clearly appreciated in the carbon EDX map obtained for the electrografted EIP-treated substrate (Figure 4c), which showed a low carbon signal in the same zones where a high-intensity iron signal was observed. In this substrate, the presence of iron was useful to define two 5 µm size-intermetallic particles.

**Figure 5:**
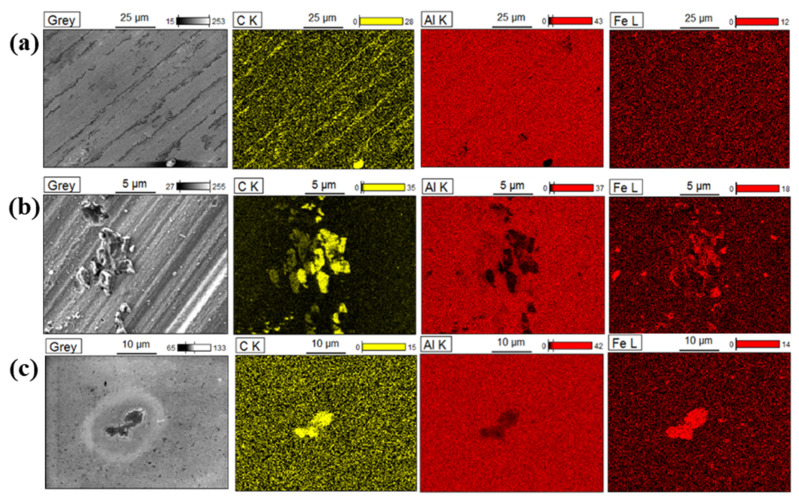
FE-SEM images and EDX maps of the 4-NBD electrografted substrates in the short potential range: (**a**) SC, (**b**) ESC and (**c**) EIP.

An FE-SEM-EDX analysis was also carried out on the modified Al-7075 surfaces electrografted by means of the short potential range. Figure 5 depicts these results, which evidenced an enhanced formation of the film on the iron-containing intermetallic particles, particularly in the case of the ESC and EIP substrates, since the highest carbon EDX signals matched the zones where the iron signals were detected. In contrast, the formation of the film on the rich Al and Al*_x_*O*_y_* zones seems to be poor, considering the low intensity of the carbon EDX signals in the regions with the higher intensities for the aluminium EDX signal. These results indicate that the application of the short potential range promotes the formation of the film on the intermetallic particles, whereas the large potential range favours the electrografting of 4-NBD on the Al-rich zones.

### 3.4. AFM Study on the Morphology of the Bare Substrates and the 4-NBD Films

**Figure 6:**
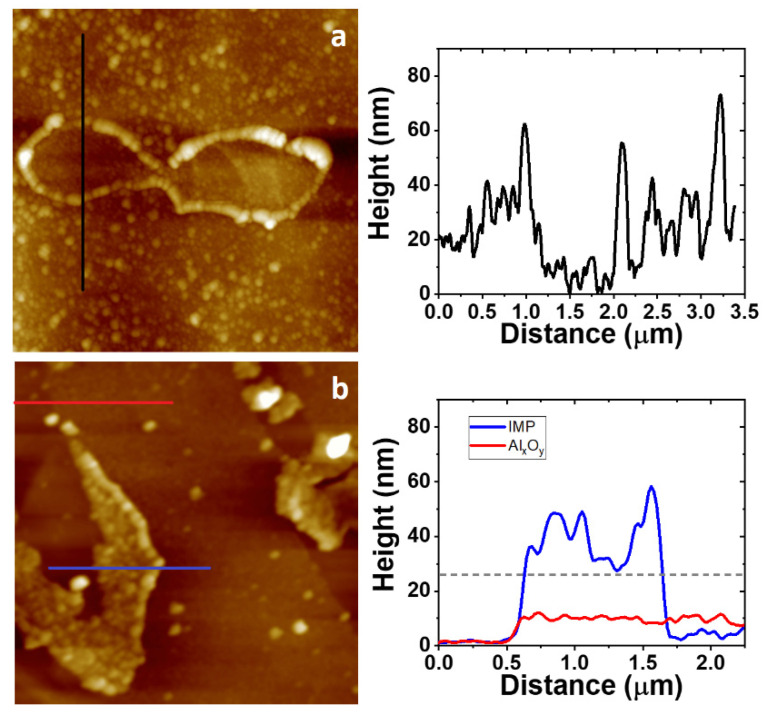
The 5.0 × 5.0 μm^2^ AFM images collected for EIP substrates cycled in a 2 mM 4-NBD containing acetonitrile (ACN) solution using the large (**a**) and the short (**b**) potential range. Cross sections taken on a representative intermetallic particle (IMP; blue line), the Al alloy region (red), or both (black) are displayed on the right panels. The estimation of the thickness range of the nitrophenylene layer formed on top of the IMPs was carried out by taking cross section profiles passing through defects or pinholes in the organic layer reaching the bare IMP surface. The dashed grey line indicates the level height for the bare IMP.

AFM images measured after the electrografting with 4-NBD are displayed in Figure 6. These images were collected for the EIP-treated Al-7075 substrates, since the roughness of the surface, after the ion-polishing procedure, is the lowest between the working substrates, namely SC, ESC and EIP, as can be seen in Figure S3 in the Supplementary Information. Consequently, minimum variations in the surface roughness due to the growth of a nanometric-sized nitrophenylene film should be detected. The AFM images in Figure 6 show the presence of a granular organic layer, i.e., the as-deposited nitrophenylene layer, when compared to that exhibited by the bare EIP Al-7075 surface. Interestingly, both FE-SEM and AFM images show that, even after thoroughly rinsing and ultrasonicating in ACN, a compact and defectless nitrophenylene layer is observed, indicating the formation of a robust organic layer chemisorbed to the Al alloy surface. Figure 6a indicates that electrografting of 4-NBD obtained by applying the large potential range mostly occurs in the Al alloy region, while the grafting reaction seems to be slightly inhibited on the intermetallic particles surface. Interestingly, an enhanced growth of the organic film could be detected at the interface between the intermetallic particle and the alloy matrix where micro-galvanic couples are developed. The presence of some cracks in this organic layer allows to estimate the thickness of the as-deposited film in the Al alloy matrix by means of cross-section profiling as displayed in Figure S4 in the Supplementary Information. The thickness of the layer ranged between 30 and 90 nm. Most importantly, the AFM images registered for the nitrophenylene layers obtained after cycling within the short potential range (Figure 6b), showed the opposite behaviour: a thin (~10 nm thick) and incomplete organic layer deposited in the Al alloy matrix, and a thick, compact and defectless nitrophenylene layer on the intermetallic particles (~15–30 nm thick).

### 3.5. PM IRRAS, Raman and XPS Characterisation of the Electrografted Films

**Figure 7:**
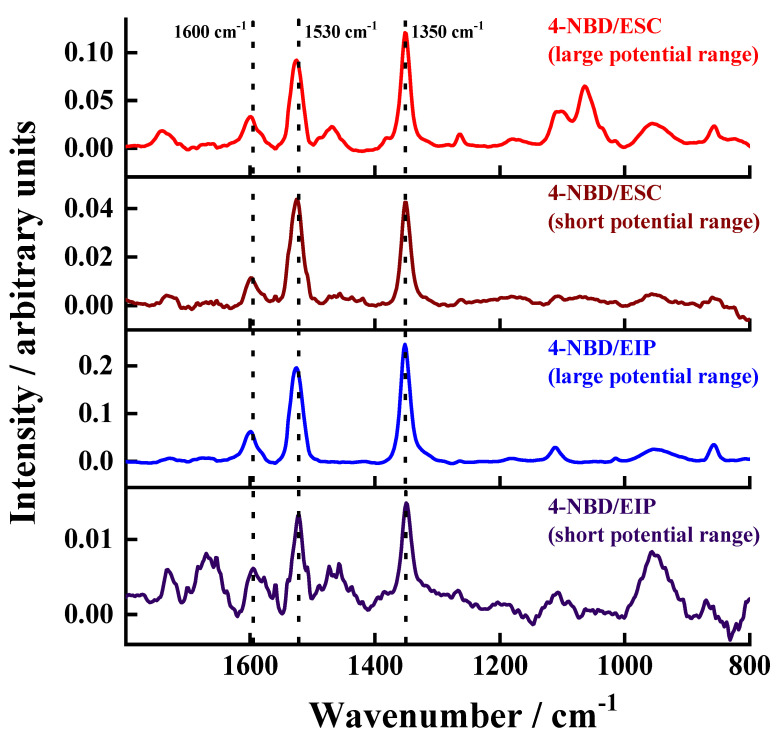
PM-IRRAS data of the 4-NBD-electrografted ESC and EIP substrates applying the large and short potential ranges.

Bearing in mind the high intensity of carbon signals observed in the EDX maps, PM-IRRAS and Raman spectroscopy characterisations were done to verify the presence of the films on ESC and EIP. In the case of the PM-IRRAS characterisation (see Figure 7), the modified substrates developed a band around 1600 cm^−1^, which corresponds to the aromatic C=C ring stretching. Moreover, two bands were observed around 1530 and 1350 cm^−1^, which correspond to the antisymmetric and symmetric stretching vibrations of NO_2_, respectively. The appearance of these bands for nitrophenylene films was already observed by Adenier et al. [33], who electrografted 4-NBD on mild steel plates for improved corrosion resistance. Hinge et al. also electrografted this molecule on stainless steel, finding the same IR bands as detected in the present work [47]. Berisha and coworkers also observed these contributions for the coupling of mixed alkyl/aryl layers on oxidised Al, obtained from the electrografting via electroreduction of an iodo-alkane in the presence of 4-NBD [44].

In addition, Raman spectroscopic characterisation proved the successful formation of the 4-NBD film on ESC and EIP-treated substrates (Figure S5 in the Supplementary Information), since intense bands around 1150, 1350, and 1600 cm^−1^ were observed, which correspond to the bending of CH groups, the stretching of NO_2_ groups and the aromatic ring stretching, respectively [31]. The G band (located at 1580 cm^−1^) represents the sp_2_ carbon system bound to the surface. According to the above analysis, it can be concluded that a successful modification of the as-treated Al alloy surfaces with nitrophenylene-based films was achieved.

**Figure 8:**
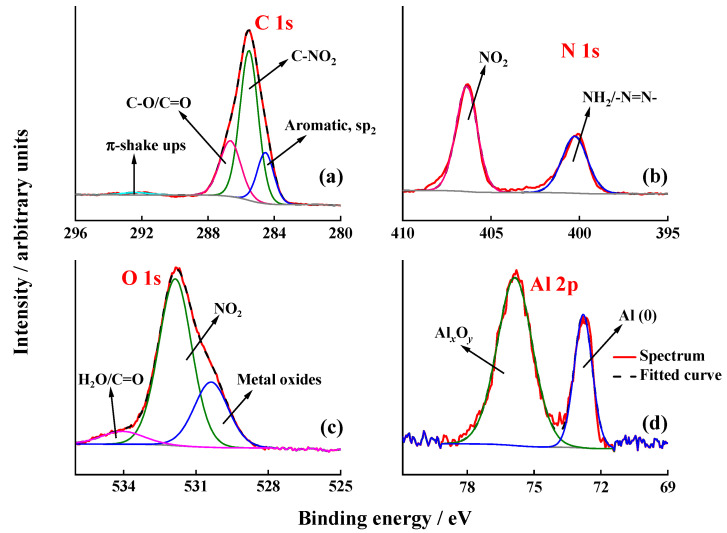
High resolution XPS spectra registered for an EIP substrate after the 4-NBD electrografting in the C 1s region (**a**), N 1s (**b**), O 1s (**c**) and Al 2p (**d**).

For the analysis of the exact chemical composition XPS data were measured, for the EIP-modified substrate, and the results are depicted in Figure 8. A detailed analysis of the C 1s spectrum (see Figure 8a) shows that 98% of the signal can be attributed to C-NO_2_ and aromatic C–H bonds, besides a broad peak at 291 eV corresponding to the π–π* transition of the aromatic ring [42]. No evidences of metal, M (Al, Fe, Cu), to C (aryl) bond were found, since no contribution at 282.4 eV could be appreciated in any of the core-level C 1s spectra. Some studies linked this contribution with the formation of Fe–C and Cu–C, respectively, although the formation of M–O–C(aryl) bonds was proposed as well [20,22]. Indeed, Hinge et al. showed that 4-NBD molecules were grafted to chromium substrates (coated with a thin layer of chromium oxide) via the formation of Cr–O–Aryl bonds [42]. Berger et al. showed a similar result for an electrodeposited ZnNi layer on steel [37]. Anyhow, the absence of the contribution at 282.4 eV in Figure 8a is not surprising given the presence of a minimum of 1.8 nm thick aluminium oxide layer. Moreover, given the extremely average surface area of intermetallic particles, a detection of corresponding M–C (Fe, Cu) bonds would not be possible by means of XPS. In this regard, Berisha et al. confirmed by means of TOF-SIMS the covalent attachment of aryl radicals to the surface of oxidised pure Al, i.e., Al–OH and Al_2_O_3_ [44]. This coupling is explained in terms of H abstraction by the aryl radical or direct attack to the oxygen in Al_2_O_3_. Figure 8b displays the N 1s core-level spectrum which can be fitted to two different contributions at 399 and 406 eV. The first one is mostly attributed to diazenyl groups that occurred after azo coupling reactions or amine groups produced by reduction of nitro moieties because of X-ray irradiation during XPS measurements. The contribution at 406 eV is commonly assigned to the NO_2_ groups present in the organic layer [39]. Figure 8c shows the high-resolution O 1s spectra registered for the electrografted 4-NBD, which exhibits a pronounced signal at 530.4 eV originating from O–Al (i.e., aluminium oxide) in the outer layer. Interestingly, an additional peak is seen at higher binding energies (533 eV), which can be assigned to oxygen in the nitro group. In the case of the high-resolution Al 2p spectra, a high contribution of the Al (0) located at 72.7 eV was detected, as expected for ion polished treated substrate, according with the characterisation performed for this EIP described in Section 3.1. In principle, XPS measurements show that nitrophenyl groups are present on the surface of EIP grafted surfaces and that metal-oxygen-carbon covalent bonding dominates the interface [42]. Considering that the outer layer of the metal surface mostly consists of aluminium oxides, a covalent attachment model consisting of Al–O–C bonding seems to be the most reasonable option.

### 3.6. Scanning Kelvin Probe Force Microscopy (SKPFM) Analysis of the Modified Surfaces

**Figure 9:**
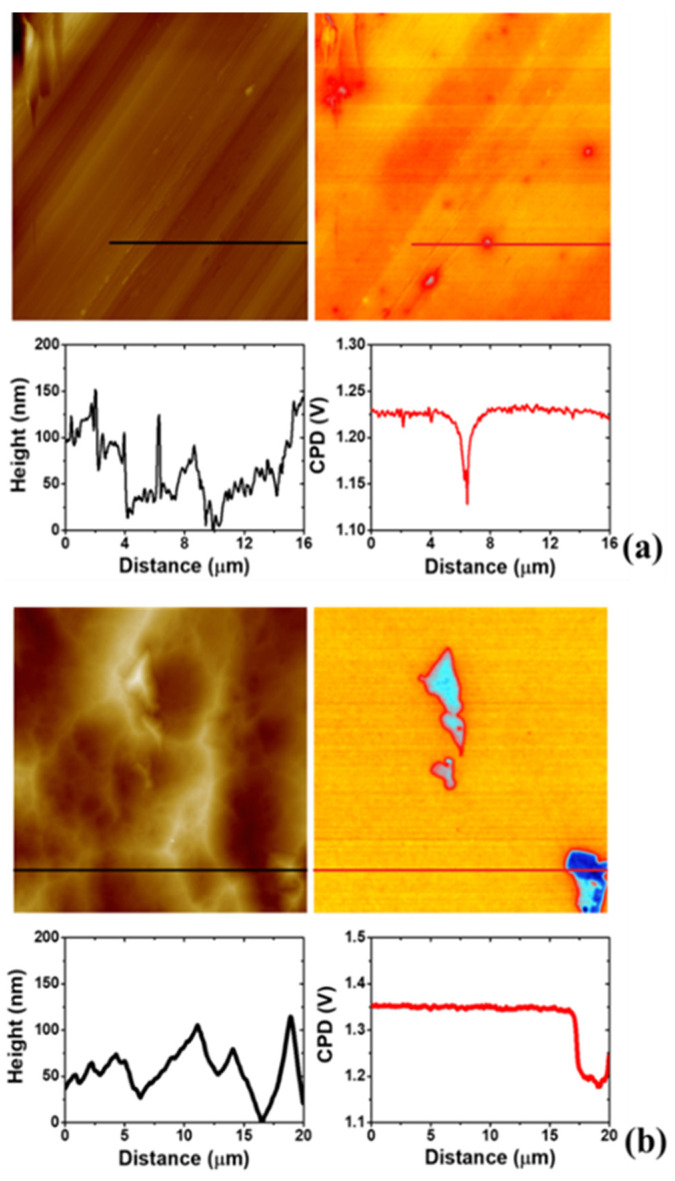
SKPFM of the ESC (**a**) and the EIP (**b**) substrates before the 4-NBD electrografting.

In order to confirm the proposed mechanism and the active sites where the 4-NBD electrografting takes place, a SKPFM analysis was carried out. Figure 9 shows the effect of the ion-polishing treatment applied on a solvent-cleaned substrate (SC, Figure 9a) to obtain an EIP surface (Figure 9b). This effect is related with the unveiling of the intermetallic particles, entities that play a key role in the electrografting of 4-NBD. In this sense, it is important to highlight that a high contact potential difference (CPD) value was obtained for the EIP substrate in a zone where no intermetallic particles are developed. This value was 1.350 V, compared with 1.225 V obtained for the SC substrate. The increase in the CPD value indicates the formation of a thinner Al oxide layer generated after the ion-polishing treatment than that of the SC substrate, an effect caused by the impact of the Ar^+^ beam on the sample surface. This trend can be justified in terms of the degree of penetration (in the range of a few nanometers) of the electromagnetic field in the direction normal (*Z*-axis) to the surface as recently pointed out by Örnek et al. [50]. That is the reason why the Volta potential exhibited by such a low noble metal like Al still appear at very positive potentials (when the tip is biased), even when it is coated by a dense and compact layer of (very noble) Al_2_O_3_, sensing then the pure Al underneath. Thus, a thicker oxide film layer would be consequently accompanied by (slightly) lower Volta potential values as shown herein. Moreover, there is a significant decrease in the CPD values (around 200 mV) in the regions where the intermetallic particles were observed by means of the AFM image, which makes evident the different composition of these particles compared with the other zones of the surface. In this regard, Birbilis et al. have determined, even for untreated Al-7075 substrates, that more than 65% of the intermetallic particles present in the Al-7075 alloys would correspond to Al_7_Cu_2_Fe [4]. The observed surface potential difference accounts for the unveiling of these intermetallic particles containing more noble metals like Fe or Cu from the Al alloy matrix, during the surface treatment [50].

**Figure 10:**
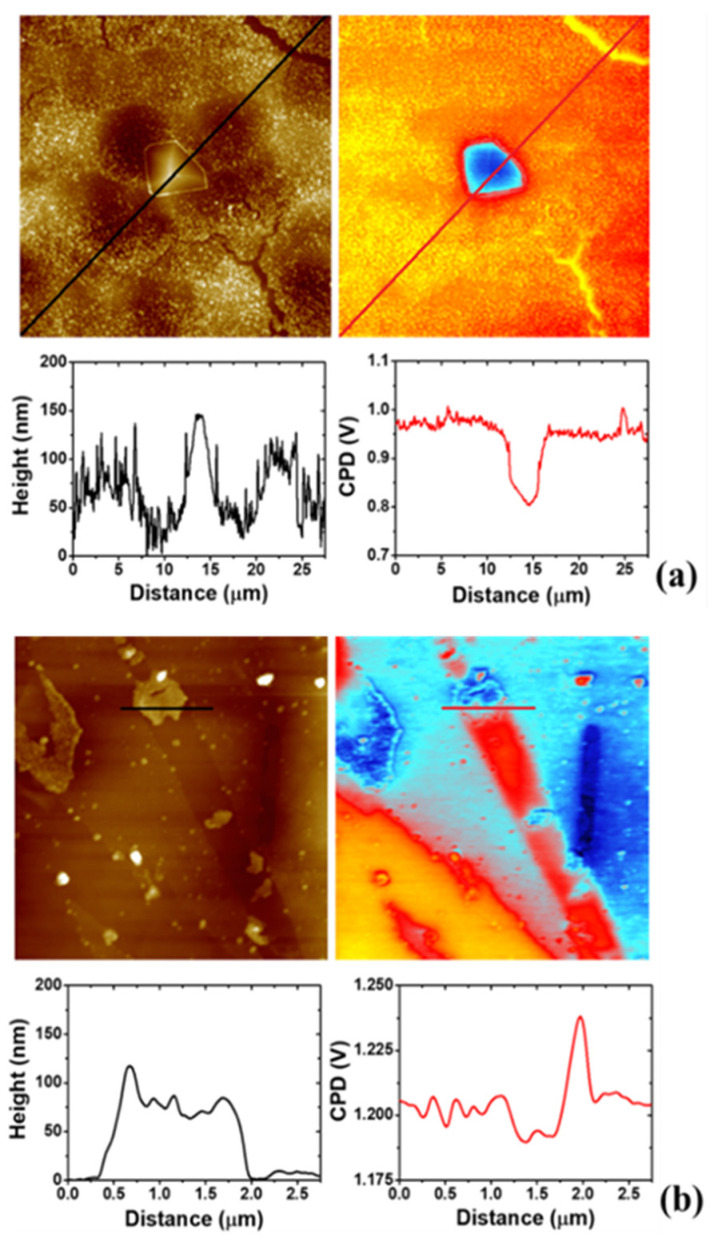
SKPFM of nitrophenylene-electrografted EIP substrates applying the large potential range (**a**) and the short potential range (**b**).

Some variations were detected when the 4-NBD molecule was electrografted to form a film, depending on the applied potential range. Figure 10a shows the result of the electrografting performed by applying the large potential range to a EIP substrate, where it is possible to appreciate a homogeneous covering of the surface with the molecule layer and a decrease in the CPD (0.975 V), in the zones where no intermetallic particles are present, in comparison with the values observed for the bare EIP substrate (1.350 V, see Figure 9b). The changes in the CPD after the formation of the nitrophenylene layer can be appreciated in more detail in Figure S6 in the Supplementary Information. In fact, at the intermetallic particles, there is a slight decrease in the surface potential difference regarding the Al alloy matrix, when compared with the same magnitude observed for the case of the unmodified EIP substrate, verifying that the use of the large potential range does not promote a significant surface modification of the intermetallic particles. However, when the short potential range is applied for electrografting (see Figure 10b), some film-defective Al alloy oxide areas identified by their high CPD values (yellow and red zones in the CPD image) which closely match those registered for untreated EIP surfaces (Figure 9) are observed. These regions are alternated with some other Al alloy oxide regions on top of which thin nitrophenylene films with a thickness of 10 nm are formed (cyan regions in the CPD image in Figure 10b). As can be deduced from the cross section profile, the presence of such thin nitrophenylene layers caused overall lower differences in the surface potential when compared to those obtained when applying the large potential range. Still, the lower CPD values (strong blue) allow to identify the intermetallic particles modified with nitrophenyl films exhibiting averaged thicknesses of 30 nm according to the cross section profile in the AFM image. From these observations it is possible to state that the deposition employing a large potential range possibly promotes the electrografting of the nitrophenylene layers mainly on the Al alloy oxides zones of the surface, whereas for a short potential range the formation of the film on the intermetallic particles is favoured.

### 3.7. Optimisation of the Growth of Nitrophenylene Films on Al-7075

**Figure 11:**
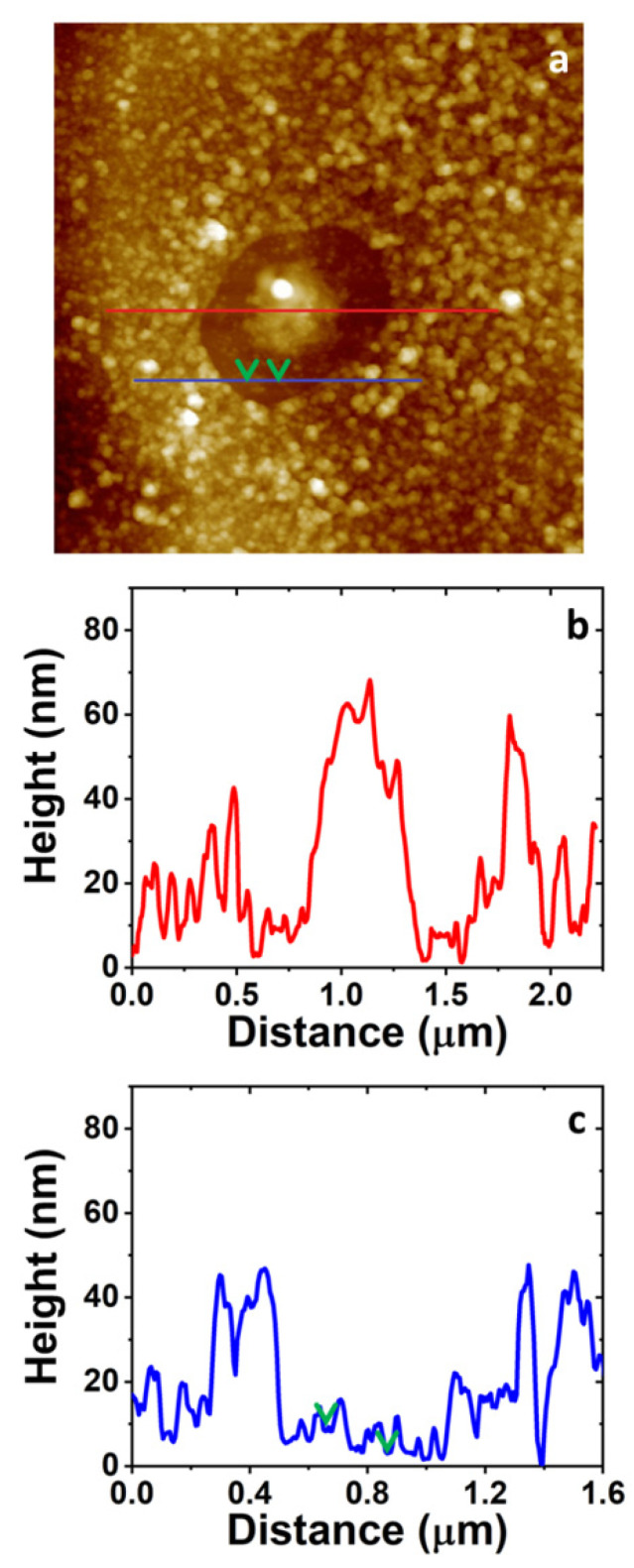
(**a**) The 3.0 × 3.0 µm^2^ AFM image of a 4-NBD electrografted EIP substrate employing first, the large potential range and secondly, the short potential range. Representative cross section of the IMP considering the middle (**b**, red line) and the outer part (**c**, blue line). Green arrows indicate the presence of a defect in the organic layer which allows for the estimation of its thickness.

Considering the above-mentioned results, a new strategy for the growth of the nitrophenylene films on the Al-7075 could be suggested as follows: EIP substrates were cycled in the 4-NBD-containing ACN solution by using first the large potential range. Then, the as-modified EIP surfaces were cycled again in the same electrolyte, but, in this case, the short potential range was applied. Following these experimental conditions, it should be possible to form the 4-NBD film on both, the Al oxide layer and the intermetallic particles, by means of the large and the short potential ranges, respectively. AFM images displayed in Figure 11a shows the formation of a thick nitrophenylene layer on the Al(oxide) alloy matrix, analogue to that shown in Figure 6a and Figure S4 in the Supplementary Information. Interestingly, unlike what is observed when either the large or the short potential range is used, the IMPs seem to be modified by a dense nitrophenylene film which is thicker in the middle of the particles while gets gradually thinner when moving from the centre of the IMP to the borders. Figure 11b displays a representative cross section profile showing the thickness of the nitrophenylene layer grown in the middle of the IMP and the surrounding Al(oxide) alloy matrix as well. In addition, at the outer regions of the IMPs, a much thinner but almost continuous layer ranging 5–10 nm thick is observed, as can be deduced from Figure 11c (see green arrows), a result that is consistent with what could be observed on the IMPs when only the large potential range was applied (Figure 6a). Some other examples of these modified IMPs are provided in Figure S7 in the Supplementary Information. The thicknesses observed for the deposited films in the different described regions can be tentatively explained as a consequence of the current density distribution caused by the characteristic morphology of the crystalline IMPs, i.e., centred apex-shaped, as can be seen in Figure S6.

## 4. Discussion

From the SEM, EDX, AFM, PM-IRRAS, Raman, XPS and SKPFM results, some conclusions can be drawn concerning the electrografting process. When this is achieved by means of applying the large potential range, the electrografting of 4-NBD is not favoured on the intermetallic particles but promoted on the Al and Al*_x_*O*_y_* regions in the Al alloy matrix. The electrografting on the intermetallic particles occurs, but it is noticeably limited in comparison to what happens in the aluminium oxide regions at potentials more negative than that of the reduction peak (−0.6 → −1.0 V vs. Ag/AgCl). Nevertheless, when the short potential range is applied, the scenario is the opposite: thin and defective nitrophenylene layer deposited on the aluminium oxide area, but compact and thick organic film grafted on the intermetallic particles. These reported phenomena can be tentatively explained as follows:

(i) The irreversible diffusion-controlled peak observed for ESC and EIP-treated Al-7075 substrates containing a thinner Al oxide layers, as shown in Figure 2b,c, can be attributed to the reduction of the diazo groups in 4-NBD to form aryl radicals in the solid–liquid interface upon release of N_2_. This would result in the covalent binding of nitrophenylene layers on the Al/Al oxide alloy matrix. This potential value, around −0.6 V (vs. Ag/AgCl), is more negative than that expected for the unveiled intermetallic particles containing more noble metals like Fe and Cu. Indeed, for the intermetallic particles the reduction peak is reported to appear at potentials even more positive than their oxidation potential [32,47]. At this point it is important to highlight that diazonium salts can be spontaneously reduced on the metals conforming the IMPs. However, this grafting is negligible compared with that generated after the application of a potential. Therefore, the main properties of the films studied in this work correspond to the films deposited via the application of a potential or a potential sweep. Furthermore, the spontaneous grafting is more favoured in aqueous/acidic conditions, which were not employed in our experiments [44,58]. Control experiences in which the as-treated Al electrodes were immersed in the grafting solution at OCP for the same time required for deaerating the ACN solution did not show noticeable modifications of the IMPs surface either by AFM or PMIRRAS (data not shown).

(ii) When the electrografting is carried out by cycling within the large potential range, i.e., (−1.0 ↔ −0.1 V (vs. Ag/AgCl)), the desorption of strongly physisorbed material from the more noble metal-containing intermetallic particles may occur. This fact is known to take place significantly by potential cycling when the cathodic limit is set close to the potential for the reduction of nitrophenyl groups to the nitrophenyl radical anion [30]. This reversible redox couple is not observed in the selected range potential for the Al alloy.

(iii) Generation of radical superoxide, O_2_^•−^, or radical hydroxide, ^•^OH, at the solid–liquid interface of the intermetallic particles is a result of the electrochemical reduction of residual oxygen in ACN. Breton and coworkers have demonstrated a potential-dependent limited growth of nitrophenylene layers on GC and PPF electrodes in the presence of oxygen. This process has been attributed to the formation of the radical superoxide at more negative potentials than the reduction of 4-NBD, i.e., −1.0 V_Ag/AgNO__3_. Thus, this radical superoxide would react with the diazo group of the 4-NBD with the subsequent formation of the nitrophenyl radical which would rapidly react with ACN, oxygen moieties or other 4-NBD molecules to produce other side products. The molecular oxygen generated after the reaction between the radical superoxide and the 4-NBD can be reduced again at the electrode surface at these very negative potentials, thus closing the catalytic cycle. In fact, thinner grafted nitrophenylene layers have been obtained in this way [25]. Moreover, iron and copper surfaces are very well known to catalyse the reduction of molecular oxygen to hydrogen peroxide or water, while their ions have been reported to take part in the Fenton reaction [59,60]. Comparatively, the overpotential for this reaction is expected to be shifted to much more negative values for the low conductive aluminium oxide layer. For clarification purposes, the Supplementary Information contains a detailed study on the electrochemical performances of Cu and Fe electrodes in ACN in the presence of molecular O_2_, showing the cyclic voltammograms for the electrografting of the 4-NBD on Cu and Fe substrates (Figure S8).

(iv) These electrochemical potentials required for the reduction of residual oxygen cannot be achieved when the phenylene layer is grafted by cycling within the short potential range, i.e., (−0.4 ↔ −0.1 V (vs. Ag/AgCl)). This would explain the progressive deposition of nitrophenylene layers on the more noble metal-containing intermetallic particles. On the other hand, on the Al alloy matrix a less effective grafting process takes place as a consequence of the reduced potential range: a limited amount of these nitrophenyl radicals can still be generated at lower potentials, as can be deduced from Figure 3b,c where low faradaic current densities can be detected close to the cathodic limit.

**Figure 12:**
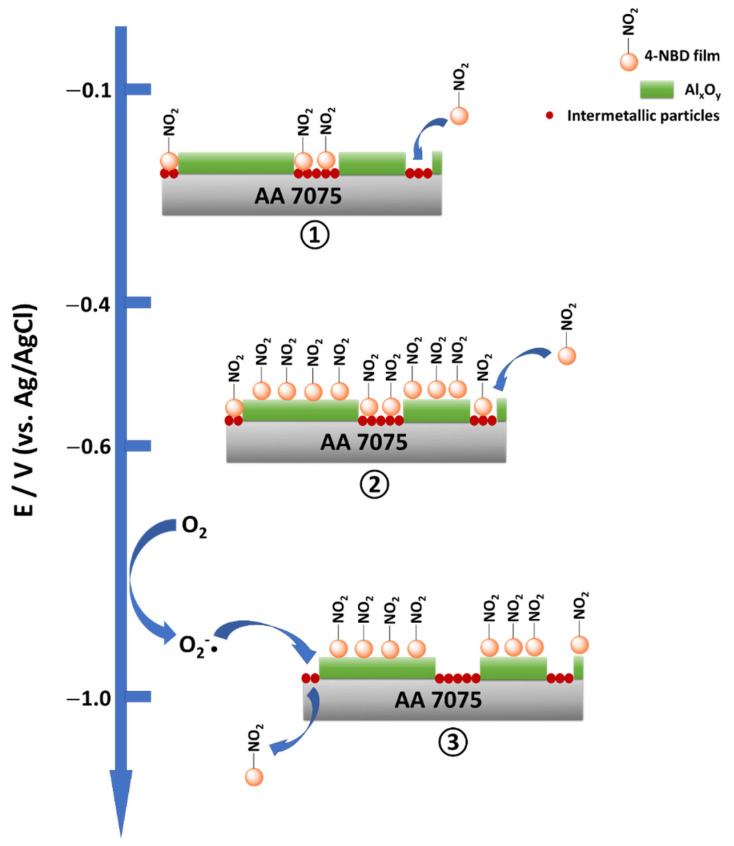
Diagram summarising the differential grafting process depending on the potential range considered.

A scheme summarising the tentative mechanism proposed for the growth of the nitrophenylene film on Al-7075 is displayed in Figure 12, although it is important to highlight that more studies are needed in order to gain a deeper understanding of the effect of the ORR on the growth of these nitro-oligophenylene films onto IMPs. Such studies are of fundamental importance for future applications concerning the corrosion resistance and adhesion properties of these Al alloys.

## 5. Conclusions

Different surface treatments, i.e., chemical etching and ion polishing were applied to Al-7075 specimens and led to define ultra-thin oxide surface layers and uncovered microscopic intermetallic phases. A minimum thickness value of 1.8 nm for the ion-polished substrates was achieved. The corresponding process analysis showed that such low oxide thickness values are critical for a successful electrografting of 4-NBD films. Furthermore, a differential behaviour concerning the composition of the arising surface defects was observed: while chemical etching promotes the unveiling of both anodic and cathodic IMPs, ion polishing favours the development of cathodic IMPs. In contrast to the films obtained for solvent cleaned surfaces, more homogeneous and thicker films were obtained for the chemically etched and ion polished substrates as confirmed by AFM, PM-IRRAS, Raman, FE-SEM/EDX, and XPS studies. By carefully choosing the applied potential range, the films could be deposited either on the oxide covered aluminium matrix, the IMP, or on both surface areas. SKPFM data proved the local deposition processes and their dependence on the applied potential range. Chemisorption of 4-NBD was identified as the most probable interfacial binding mechanism. This anchoring of an ultra-thin organic film to a high strength Al-alloy is a promising prospect of an efficient way to improve the corrosion resistance and adhesive properties of such alloys.

## Figures and Tables

**Table 1:** Results of XPS elemental analysis in atomic percentage (at-%) for the studied substrates: solvent cleaning (SC), chemical etched-solvent cleaning (ESC) and chemical etched-ion polished (EIP).

Substrate	Atomic Percentage per Element (at-%)							
	C 1s	N 1s	O 1s	Al 2p	Zn 2p_3/2_	Fe 2p_3/2_	Cu 2p_3/2_	Mg 2s
SC	44.8	1.3	32.3	21.5	0.2	-	-	-
ESC	15.9	0.3	45.7	37.2	0.5	-	0.2	0.1
EIP	19.4	0.2	41.1	38.7	0.1	0.2	0.2	0.1

**Table 2:** Peak areas and parameters employed to calculate the thickness *d* of the Al oxide layers developed after applying the different surface treatments.

Substrate	*I_Ox/_*Area%	*I_M/_*Area%	*λ_Ox/_*nm	*λ_M/_*nm	*N_Ox_*	*N_M_*	*sin θ*	*d/*nm
SC	81.3	18.7	2.8	2.6	1.0	1.5	0.5	2.7
ESC	77.9	22.1	2.8	2.6	1.0	1.5	0.5	2.5
EIP	65.1	34.9	2.8	2.6	1.0	1.5	0.5	1.8

## Data Availability

The data presented in this study are available on request from the corresponding author.

## Supplementary Materials

The following are available online at https://www.mdpi.com/article/10.3390/nano11040894/s1, Figure S1. SKPFM images of an Al-Si-Au patterned grid (collected when the Pt-coated tip is biased): topographic (a) and contact potential difference (b) images. Right panels show the corresponding cross section profiles, Figure S2. FE-SEM images and EDX maps of the bare substrates after the (a) SC, (b) ESC and (c) EIP treatments, Figure S3. Representative 10 × 10 µm^2^ AFM images collected from Al-7075 substrates after different surface treatments: SC (a), ESC (b), and EIP (c). The corresponding cross section profiles are displayed on the right panels, Figure S4. The 5.0 × 5.0 µm^2^ AFM image collected from an EIP-modified Al-7075 substrates after cycling in a 2 mM 4-NBD-containing ACN solution, left panel. The corresponding cross section profile showing the thickness of the nitrophenylene layer is displayed on the right panel, Figure S5. Raman spectra of the 4-NBD-electrografted ESC and EIP substrates applying the large and short potential ranges, Figure S6. The 5.0 × 5.0 µm^2^ SKPFM images collected from an EIP-modified Al-7075 substrate after cycling in a 2 mM 4-NBD-containing ACN solution within the large potential range, Figure S7. The 2.0 × 2.0 µm^2^ 3D AFM images collected from an EIP-modified Al-7075 substrates after cycling in a 2 mM 4-NBD-containing ACN solution as indicated in Section 3.7, Figure S8. Cyclic voltammograms registered at 0.05 V·s^−1^ for freshly polished polycrystalline Cu (upper panels) and Fe (lower) in a in a deaerated 2 mM 4-NBD containing 0.1 M TTBAFB4 ACN solution (a and c) and in a 0.1 M TTBAFB4 ACN solution (b and d). Table S1. Carbon and nitrogen composition determined by EDX of the electrografted 4-NBD films applying the large and short potential ranges.
