# Supplementary material for: Electrografting of 4-Nitrobenzenediazonium Salts on Al-7075 Alloy Surfaces—The Role of Intermetallic Particles
Source: Nanomaterials (Basel). 2021 Mar 31;11(4):894. doi: 10.3390/nano11040894 (PMC8067352; doi:10.3390/nano11040894)
Supplement: Supplementary file 1 [file nanomaterials-11-00894-s001.pdf]

nanomaterials

Article

Electrografting of 4-Nitrobenzenediazonium Salts on Al-7075 Alloy Surfaces—The Role of Intermetallic Particles

Jiangling Su [1], Juan Carlos Calderón Gómez [1,*], Guido Grundmeier [1] and Alejandro González Orive [1,2,*]

[1] Department of Technical and Macromolecular Chemistry, Paderborn University, Warburger Str. 100, 33098 Paderborn, Germany; sujl@mail.uni-paderborn.de (J.S.); guido.grundmeier@uni-paderborn.de (G.G.)
[2] Department of Chemistry, Materials and Nanotechnology Institute, University of La Laguna, 38200, San Cristóbal de La Laguna, Spain
* Correspondence: jccalderong@gmail.com (J.C.C.G.); agorive@ull.edu.es (A.G.O.) Tel.: Tel: +49 5251 60-5746 (J.C.C.G.); +34-922318020 (A.G.O.)

Figure S1 displays a SKPFM image collected for a patterned reference sample containing regular Al, Si, and Au domains (purchased from Bruker) measured with an analogue Pt tip under the same experimental condition used for the characterization of the Al alloy.

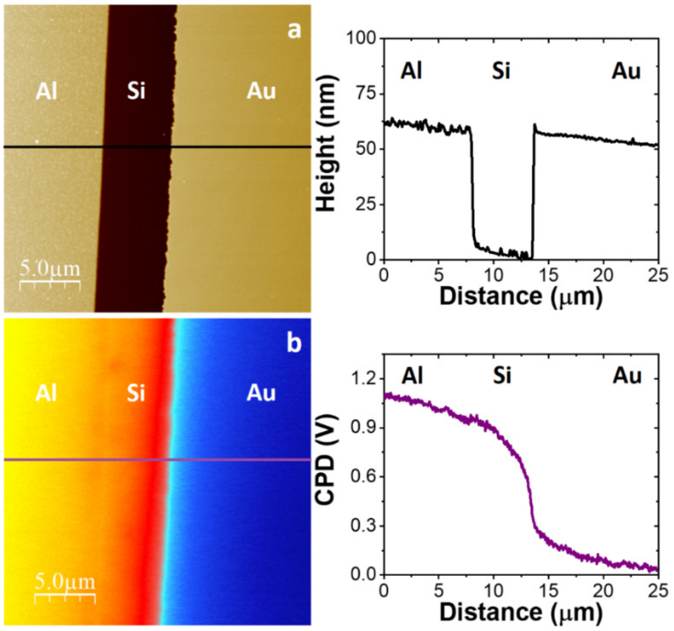

Figure S1. SKPFM images of an Al-Si-Au patterned grid (collected when the Pt-coated tip is biased): Topographic (**a**) and contact potential difference (**b**) images. Right panels show the corresponding cross section profiles.

The Figure S2 depicts the SEM-EDX images of the bare substrates before the deposition of the 4-nitrobenzenediazonium films deposition. Some Fe intermetallic particles were observed in the studied substrates.

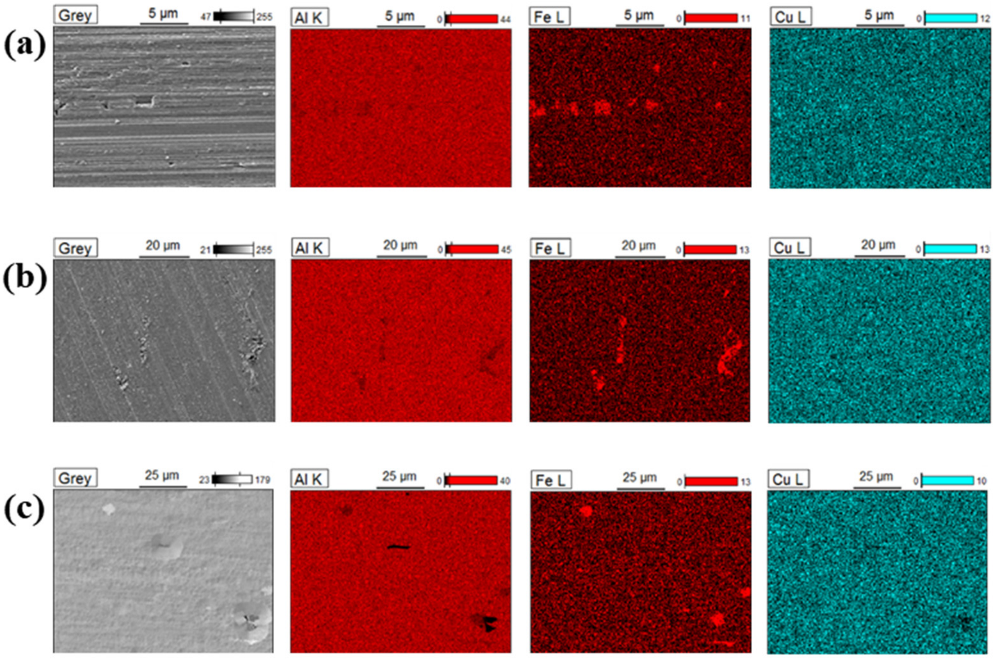

Figure S2. FE-SEM images and EDX maps of the bare substrates after the (**a**) SC, (b) ESC and (**c**) EIP treatments.

The Figure S3 shows the AFM images and cross-section profiles of the Al-7075 substrates after the different applied surface treatments. As described in the manuscript, the EIP substrate displayed the lowest roughness, a fact explained from the ion-polish treatment applied on this surface.

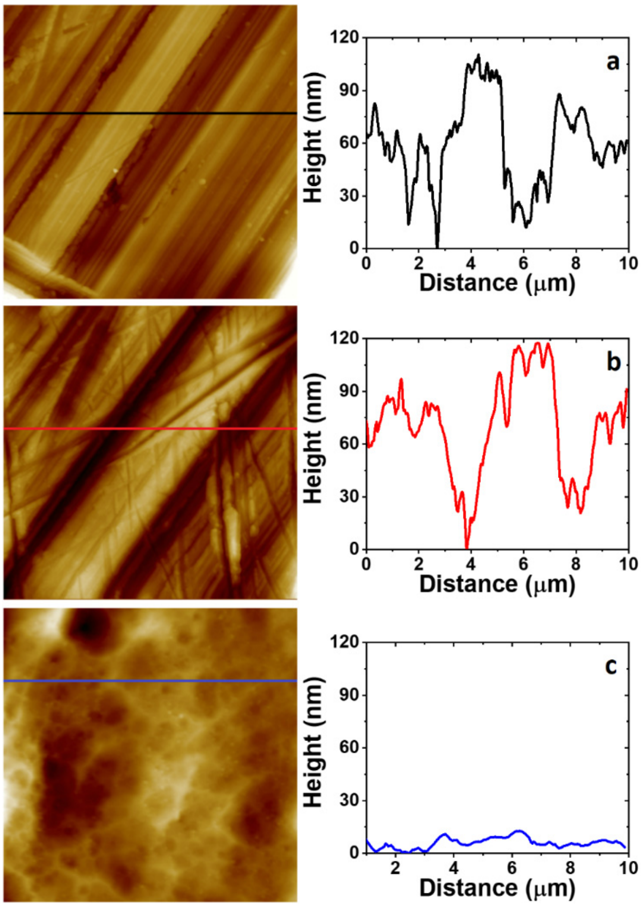

Figure S3. Representative 10 × 10 μm² AFM images collected from Al-7075 substrates after different surface treatments: SC (**a**), ESC (**b**), and EIP (**c**). The corresponding cross section profiles are displayed on the right panels.

The Table S1 shows the C and N composition of the studied 4-NBD-electrografted substrates, after applying the large and the short potential ranges. This composition has been determined by EDX analysis and shows higher nitrogen and carbon signals for the ESC and EIP substrates, in comparison with those observed for the SC substrate. This fact evidenced the successful formation of the film on ESC and EIP, due to the increase in its activity after the applied chemical treatments described in the section 2.1 of the manuscript.

Table S1. Carbon and nitrogen composition determined by EDX of the electrografted 4-NBD films applying the large and short potential ranges.

Substrate	Large potential range		Short potential range	
	wt % C	wt % N	wt % C	wt % N
SC	1.00	0.57	0.86	0.00
ESC	10.57	1.74	4.24	0.96
EIP	8.20	1.40	0.76	0.65

The Figure S4 depicts the AFM images obtained for the electrografted 4-NBD films deposited on an EIP substrate after applying the large potential range. From the cracks present in this organic layer, the thickness of the as-deposited film was estimated. This value is between 30 and 90 nm.

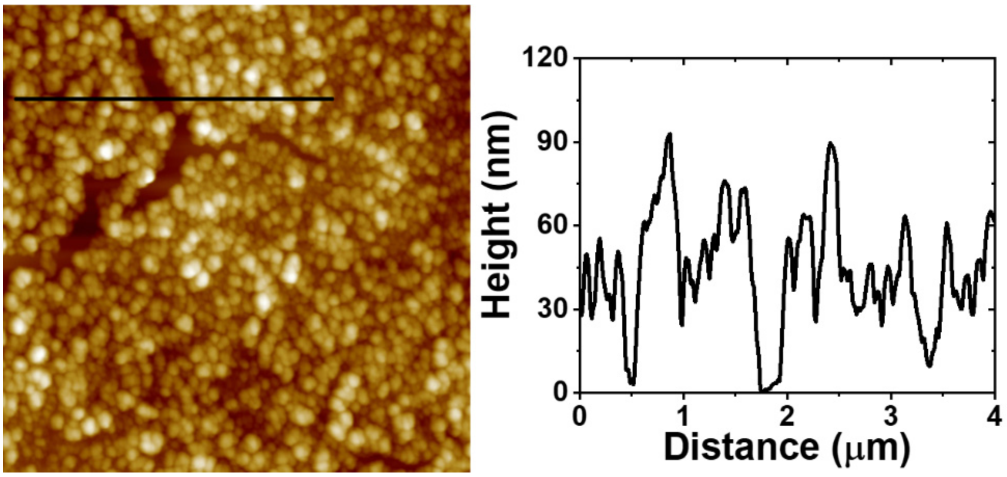

Figure S4. 5.0 × 5.0 μm² AFM image collected from an EIP-modified Al-7075 substrates after cycling in a 2 mM 4-NBD-containing ACN solution, left panel. The corresponding cross section profile showing the thickness of the nitrophenylene layer is displayed on the right panel.

The Figure S5 shows the Raman spectra for the 4-NBD electrografted films deposited on the ESC and the EIP substrates. The assignments for these bands are detailly described in the section 3.5 of the manuscript.

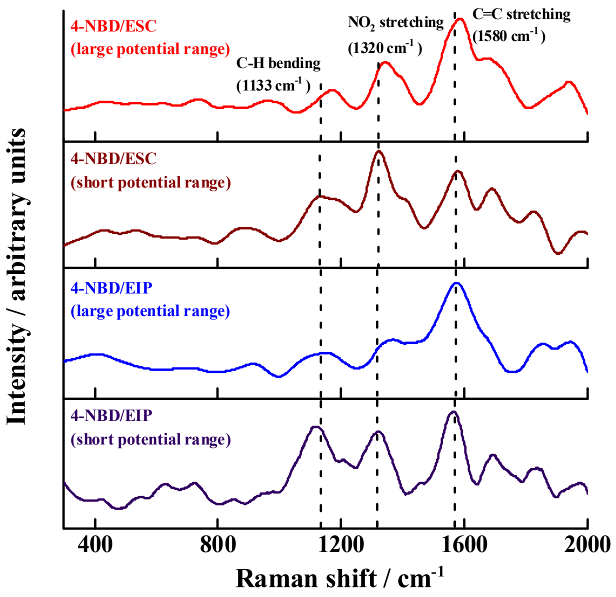

Figure S5. Raman spectra of the 4-NBD-electrografted ESC and EIP substrates applying the large and short potential ranges.

In the Figure S6, the SKPFM images obtained for the films deposited on the EIP substrate applying the large potential range are presented, where it is possible to appreciate both the preferential formation of the film on the Al_xO_y surface and the characteristic morphology of the crystalline IMPs, i.e. centred apex-shaped, as described in the sections 3.6 and 3.7, respectively.

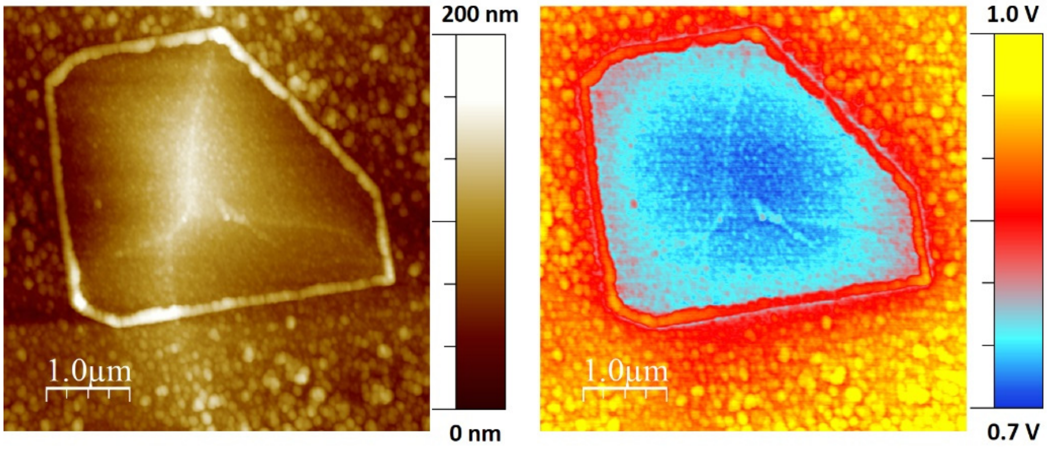

Figure S6. 5.0 × 5.0 μm^2 SKPFM images collected from an EIP-modified Al-7075 substrate after cycling in a 2 mM 4-NBD-containing ACN solution within the large potential range.

The Figure S7 displays the 3D AFM images of the 4-NBD films electrografted on an EIP substrate applying both, the large and short potential ranges. The images show that there is a lack of film in the borders of the intermetallic particle, which has been explained from the interface formed between the IMP and the Al_xO_y.

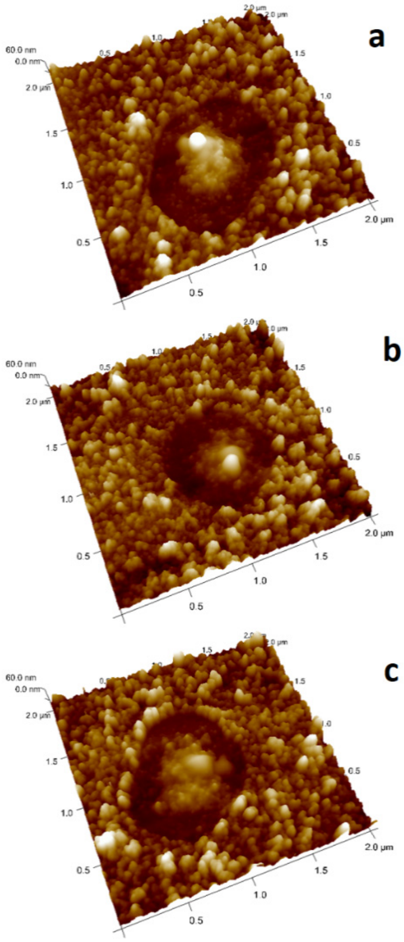

Figure 7. 2.0 × 2.0 μm^2 3D AFM images collected from an EIP-modified Al-7075 substrates after cycling in a 2 mM 4-NBD-containing ACN solution as indicated in section 3.7.

Since Cu and Fe are the major components in the cathodic intermetallic articles as shown by FE-SEM/EDX and SKPFM analysis, CV measurements have been carried out on freshly polished polycrystalline Cu and Fe electrodes to clarify the mechanism for the differential electrografting process of nitrophenylene films occurred onto Al-7075 surfaces. Figure SI8a shows the cyclic voltammograms registered for the Cu substrate in a deaerated solution 0.1 M $TTBAFB_4$ in ACN containing 2 mM 4-NBD. The characteristic irreversible wave corresponding to the reduction of the diazo group is expected to occur at potentials even more positive than the oxidation of the metallic surface itself, especially in the case of iron [1–3]. For that reason, we have consequently -constrained the positive limit for the potential range considered herein. An electrochemical redox couple with the cathodic wave appearing at −1.6 $V_{Ag/AgCl}$ and the anodic one at −1.0 $V_{Ag/AgCl}$ can be observed, which is attributed to the electrochemical response of the nitro groups present in the grafted nitrophenylene layer with their corresponding reduction to nitro radical anion and its subsequent oxidation, respectively [4–5]. Interestingly, when a freshly polished copper electrode is cycled within the same potential range, but in the absence of 4-NBD for a non-deaerated 0.1 M $TTBAFB_4$ in ACN, an irreversible diffusion-controlled voltammetric peak centered at −1.3 $V_{Ag/AgCl}$ can be observed in the first voltammogram, which can be assigned to the reduction of molecular oxygen O_2 to the superoxide radical anion $O_2^{\bullet -}$, as can be seen in the Figure SI8b. A similar range for this reaction has been recently reported by Breton and co-workers for a glassy carbon electrode [6]. The current densities associated with this peak decrease after each cycle, as can be seen in Figure S8b, which is indicative of the depletion of O_2 at the solid-liquid interface during successive scans. This

would account for an active electroreduction of molecular oxygen occurring in this range of negative applied potentials.

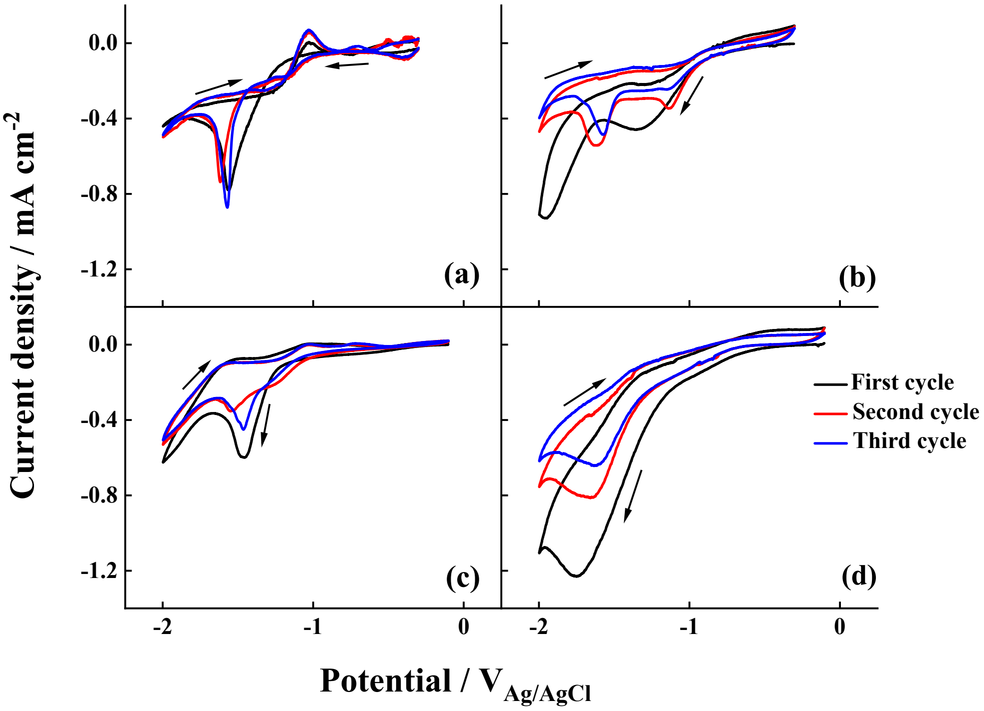

Figure S8. Cyclic voltammograms registered at 0.05 V·s^{-1} for freshly polished polycrystalline Cu (upper panels) and Fe (lower) in a in a deaerated 2 mM 4-NBD containing 0.1 M TTBAFB$_4$ ACN solution (**a** and **c**) and in a 0.1 M TTBAFB$_4$ ACN solution (**b** and **d**).

A similar behavior was found for the polycrystalline iron electrode, Figure S8c. With a cathodic wave appearing at −1.4 $V_{Ag/AgCl}$ and the anodic one at −0.9 $V_{Ag/AgCl}$ attributable to the electrochemical response of the nitro groups in the grafted film. In parallel, the diffusion-controlled irreversible wave at −1.7 $V_{Ag/AgCl}$, together with the preceding shoulder at −0.9 $V_{Ag/AgCl}$, (see Figure S8d) is attributed to the reduction of oxygen.

As previously stated, the generation of the superoxide radical has been related to the inhibition of the growth of multilayered nitrophenylene films onto conductive electrodes [6]. Similarly, during the formation of nitrophenylene films on Al-7075 by cycling within the large potential range, the very negative potentials applied herein would be responsible for the generation of superoxide radical from residual oxygen and water in the ACN solution at the solid-liquid interface of the cathodic intermetallic particles (mostly formed by Fe and Cu). In this regard, a synergistic effect of ferrous ion and copper oxide for the generation of this radical superoxide has been reported by Zhang et al [7,8]. This would result in the subsequent inhibition of the film growth onto these particles, while no significant impact might be observed on the film electrodeposited on the Al oxide matrix.

References

1. Adenier, A.; Combellas, C.; Kanoufi, F.; Pinson, J.; Podvorica, F.I. Formation of polyphenylene films on metal electrodes by electrochemical reduction of benzenediazonium salts. *Chem. Mater.* **2006**, *18*, 2021–2029.
2. Adenier, A.; Bernard, M.C.; Chehimi, M.M.; Cabet-Deliry, E.; Desbat, B.; Fagebaume, O.; Pinson, J.; Podvorica, F. Covalent modification of iron surfaces by electrochemical reduction of aryldiazonium salts. *J. Am. Chem. Soc.* **2001**, *123*, 4541–4549.
3. Hurley, B.L.; McCreery, R.L. Covalent bonding of organic molecules to Cu and Al alloy 2024 T3 surfaces via diazonium ion reduction. J. Electrochem. Soc. 2004, *151*, B252–B259.
4. Berger, F.; Delhalle, J.; Mekhalif, Z. Hybrid coating on steel: ZnNi electrodeposition and surface modification with organothiols and diazonium salts. *Electrochim. Acta* **2008**, *53*, 2852–2861.

5. Hinge, M.; Ceccato, M.; Kingshott, P.; Besenbacher, F.; Uttrup Pedersen, S.; Daasbjerg, K. Electrochemical modification of chromium surfaces using 4-nitro-and 4-fluorobenzenediazonium salts. *New J. Chem.* **2009**, *33*, 2405–2408.
6. Pichereau, L.; López, I.; Cesbron, M.; Dabos-Seignon, S.; Gautier, C.; Breton, T. Controlled diazonium electrografting driven by overpotential reduction: a general strategy to prepare ultrathin layers. *Chem. Comm.* **2019**, *55*, 455–457.
7. Zhang, Y.; Fan, J.; Yang, B.; Huang, W.; Ma, L. Copper–catalyzed activation of molecular oxygen for oxidative destruction of acetaminophen: the mechanism and superoxide-mediated cycling of copper species. *Chemosphere* **2017**, *166*, 89–95.
8. Zhang, Y.; Fan, J.; Yang, B.; Ma, L. Synergistic effect of ferrous ion and copper oxide on the oxidative degradation of aqueous acetaminophen at acid conditions: a mechanism investigation. *Chem. Eng. J.* **2017**, *326*, 612–619.
